# Protective Effects Proanthocyanidin Nanoliposome Freeze‐Dried Powder on Oxidative Injury in a p31‐43 Induced Celiac Disease Cell Model

**DOI:** 10.1002/fsn3.71000

**Published:** 2025-09-26

**Authors:** Zhou Ning, Chen Linlin, Zhang Xing, He Kunmiao, Xu Tiantian, Wang Hanshuo, Sun Jie, Yu Miao, Ren Hongtao, Wang Na

**Affiliations:** ^1^ College of Food Science and Technology Henan Agricultural University Zhengzhou China; ^2^ Key Laboratory of Nutrition and Healthy Food Zhengzhou China; ^3^ Long Hu Laboratory Zhengzhou China; ^4^ College of Life Sciences Qingdao University Qingdao China; ^5^ Institute of Food and Processing Liaoning Academy of Agricultural Sciences Shenyang China; ^6^ International Joint Research Center of National Animal Lmmunology, College of Veterinary Medicine Henan Agricultural University Zhengzhou China

**Keywords:** anti‐inflammatory, antioxidant, celiac disease, nanoliposomes, proanthocyanidins

## Abstract

Proanthocyanidins play a crucial role in celiac disease (CD) through their antioxidant and anti‐inflammatory properties, effectively regulating oxidative stress and inflammatory responses. However, the poor stability and low bioavailability of proanthocyanidins significantly limit their therapeutic applications. The aim of this study was to investigate the effects of lyoprotectants on proanthocyanidin nanoliposome freeze‐dried powder (PCNL‐FD), characterize its structural properties, evaluate its in vitro antioxidant capacity and explore its protective effects in a celiac disease cell model. The addition of 4% trehalose as a lyoprotectant effectively maintained the encapsulation efficiency and redispersion stability of PCNL‐FD. FTIR and TEM analyses confirmed the successful incorporation of proanthocyanidins into the phospholipid bilayer. In vitro antioxidant assays showed that PCNL‐FD exhibited significantly higher DPPH, ABTS, and hydroxyl radical scavenging activities compared to free proanthocyanidins. In the p31‐43 peptide‐induced celiac disease Caco‐2 cell model, PCNL‐FD significantly reduced oxidative stress by decreasing ROS and MDA levels while increasing GSH levels and antioxidant enzyme activities (SOD and CAT). The formulation showed anti‐inflammatory effects by inhibiting pro‐inflammatory cytokines (TNF‐α, IL‐6) and promoting anti‐inflammatory cytokines (IL‐4, IL‐10). In addition, PCNL‐FD activated the Keap1/Nrf2 signaling pathway and upregulated the expression of NQO‐1 and HO‐1. This nanoliposomal delivery system effectively overcame the bioavailability and stability limitations of free proanthocyanidins, providing new insights into the management of oxidative stress in inflammatory diseases such as celiac disease.

## Graphical abstract

Proanthocyanidin nanoliposome freeze‐dried powder (PCNL‐FD) with 4% trehalose maintained excellent stability and encapsulation efficiency, demonstrating enhanced antioxidant activity compared to free proanthocyanidins. In a celiac disease cell model, PCNL‐FD significantly reduced oxidative stress by decreasing ROS and MDA while increasing GSH and antioxidant enzyme activities, simultaneously inhibiting pro‐inflammatory cytokines and promoting anti‐inflammatory ones. This nanoliposomal delivery system effectively overcame bioavailability limitations of proanthocyanidins by activating the Keap1/Nrf2 pathway, offering new potential for managing oxidative stress in celiac disease.

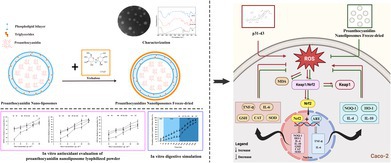

## 1 Introduction

Celiac Disease (CD) is an allergic entropathy occurring in individuals with HLA‐DQ2 or HLA‐DQ8 genes, affecting 1.4% of the global population (Rostami‐Nejad et al. 2014). The disease manifests through intestinal inflammation, villous atrophy, and crypt hyperplasia (Abadie et al. 2020). The α‐gliadin peptide p31‐43, derived from gluten protein hydrolysis, activates TG2 and triggers inflammation via CD4^+^ Th1 cell‐mediated immune responses (Bayardo et al. 2012; Cui et al. 2022; Shan et al. 2002). While a gluten‐free diet (GFD) is the primary treatment, maintaining strict adherence significantly impacts patients' quality of life. Intestinal inflammation promotes ROS production, which can be mitigated by natural antioxidant compounds through inhibition of pro‐oxidative enzymes (Sahoo et al. 2023). Therefore, developing bioavailable natural antioxidant formulations presents a promising approach for improving CD symptoms.

Proanthocyanidins (PC) are natural polyphenolic compounds composed of varying numbers of flavan‐3‐ol monomers, such as catechin, epicatechin, and gallocatechin (Qi et al. 2023). They possess diverse biological properties, including antioxidant (Chang et al. 2020), anti‐inflammatory (Wang, Cui, Xu, et al. 2022), anti‐cancer (Katiyar et al. 2017), and anti‐aging effects (Liu, Zhang, et al. 2025) (Xu et al. 2021). In Intervention for CD, Peanut skin‐derived PC alleviated oxidative stress in vitro in CD models induced by gluten‐digested peptides via modulation of the SIRT1/NRF2 pathway (Wang, Cui, Xu, et al. 2022). Grape seed PC were shown to enhance antioxidant enzyme defenses, such as SOD activity in the colon (Wang et al. 2011). Furthermore, cocoa extracts rich in proanthocyanidin B_2_ could suppress inflammation in CD Caco‐2 cell models by downregulating IFN‐γ and gliadin peptide p31‐43‐induced TG2 and IL‐15 expression (Kramer et al. 2019). However, PC are inherently unstable and highly sensitive to external environmental factors, such as temperature, light, metal ions, and pH, which significantly reduce their antioxidant efficacy (Li, Zhang, et al. 2024). To mitigate the degradation of PC, enhance their solubility, and improve their antioxidant activity, they can be encapsulated into nanocarriers to form proanthocyanidin nanoliposomes (PC‐NL) (Lv et al. 2023). Nanoliposomes, which have a structure similar to biological membranes (Pan et al. 2024), are capable of encapsulating hydrophilic compounds in their aqueous core and lipophilic compounds in their outer bilayer (Riccardi et al. 2024). They are widely utilized in drug delivery systems. However, nanoliposomes face significant stability challenges during storage, including lipid particle aggregation, phospholipid hydrolysis, and oxidative degradation (Sharma and Sharma 1997). To address these issues, freeze‐drying technology can be employed to remove water from the system, thereby preserving the stability of nanoliposomes and maintaining the bioactivity of encapsulated compounds (Varshosaz et al. 2012). Nevertheless, the freeze‐drying process converts water into ice crystals, which may compromise the stability of rehydrated nanoliposomes, causing changes in particle size, disruption of lipid membrane integrity, leakage of encapsulated materials, and reduced bioactivity of the delivered substances (Jiang et al. 2023; Yu et al. 2021). To prevent aggregation of nanoparticles after freeze‐drying and to ensure redispersibility following freeze–thaw cycles, cryoprotectants such as sorbitol, mannitol, and trehalose can be added during the freeze‐drying process. These cryoprotectants stabilize the lipid bilayer of nanoliposomes (Varshosaz et al. 2012) and protect the structural integrity of liposomes (Susa et al. 2021), thereby improving the stability of PC‐NL freeze‐dried powders.

Previous studies have demonstrated the significant potential of liposomes in alleviating intestinal inflammation and oxidative damage. Anthocyanin‐3‐glucoside nanoliposomes have been demonstrated to significantly reduce pro‐inflammatory cytokine expression in LPS‐induced intestinal inflammation using a Caco‐2/RAW264.7 co‐culture model (Yang et al. 2022). Quercetin nanoliposomes were proven effective in mitigating oxidative damage in a 3D Caco‐2 cell model induced by hydrogen peroxide (H_2_O_2_) (Liu, Zhou, et al. 2025). Novel curcumin‐based nanoliposomes modified with pectin, whey protein isolate, and hyaluronic acid have been shown to effectively improve cell survival and alleviate H_2_O_2_‐induced oxidative damage (Zhang et al. 2024). However, the protective effects of proanthocyanidin nanoliposome freeze‐dried powder (PCNL‐FD) against CD‐related inflammation and oxidative damage remain unexplored.

PC shows significant potential in treating CD due to its excellent antioxidant and anti‐inflammatory properties; however, its poor stability and low bioavailability limit applications. The study commenced with the screening of optimal freeze‐drying protectants and their respective concentrations, the optimization of preparation processes, and the subsequent characterization of PCNL‐FD utilizing FTIR and TEM. The in vitro antioxidant activity of PCNL‐FD was evaluated through DPPH, ABTS, and hydroxyl radical scavenging assays, and its stability was assessed under simulated digestive conditions. To investigate the mechanism of PCNL‐FD, we established a p31‐43‐induced Caco‐2 CD cell model, systematically evaluated the effects of PCNL‐FD on cellular oxidative stress levels and inflammatory factor expression, and explored its molecular mechanism of antioxidant and anti‐inflammatory actions through the Keap1/Nrf2 signaling pathway.

## 2 Materials and Methods

### 2.1 Materials

Proanthocyanidins and Proanthocyanidins standards were purchased from Solarbio (Beijing, China). Soybean lecithin (purity > 90%) and cholesterol (purity > 98%) were obtained from Aladdin (Shanghai, China). Trehalose (for injection) was provided by Aivotomed Pharmaceutical Technology Co. Ltd. (Shanghai, China), while mannitol was purchased from Beijing Fenglijingqiu Pharmaceutical Co. Ltd. (Beijing, China). 1,1‐Diphenyl‐2‐picrylhydrazyl (DPPH, purity > 97%) was acquired from TCI Development Co. Ltd. (Shanghai, China). Pepsin (10 FIP‐U/mg) derived from porcine gastric mucosa and pancreatic lipase (1000–2000 units/mg) derived from porcine pancreas were purchased from Sigma‐Aldrich (Shanghai, China). Mucin (type II), derived from porcine mucosal digestion, was sourced from Meilunbio (Dalian, China). Sensitizing peptide p31‐43 was obtained from Sangon Biotech (Shanghai, China). Test kits for GSH, MDA, SOD, GSH/GSSG, CAT, IL‐4, IL‐10, TNF‐α, and IL‐6 were procured from Nanjing Jiancheng Bioengineering Institute (Jiangsu, China). DMEM/F‐12 medium was supplied by Thermo Fisher Scientific Inc. (Shanghai, China), and fetal bovine serum (FBS) was purchased from OriCell (Guangzhou, China). Primary antibodies against β‐actin, Keap1, Nrf2, NQO‐1, and HO‐1 were obtained from Proteintech (Wuhan, China), and HRP‐conjugated secondary antibodies (rabbit anti‐mouse) were also purchased from Proteintech (Wuhan, China). Unless otherwise specified, all other chemicals were analytical‐grade reagents and were purchased from Sinopharm Chemical Reagent Co. Ltd. (Shanghai, China) and Macklin (Shanghai, China).

### 2.2 Preparation of PCNL‐FD

#### 2.2.1 Preparation of PC‐NL

PC‐NL was prepared using a modified reverse‐phase evaporation method described by Fan et al. (2024) and Chen et al. (2022), incorporating ultrasonic (Yun et al. 2023) and microwave (Shah et al. 2016) techniques. A total of 0.618 g of soybean lecithin and cholesterol at a mass ratio of 7:1 was weighed and mixed with 5 mL of a PC solution at a concentration of 0.6 mg/mL. The volume was adjusted to 20 mL with anhydrous ethanol. Using an ultrasonic‐microwave‐assisted extraction system (CW‐2000, Shanghai Xintuo Analytical Instrument Technology Co, CN), the mixture was sonicated at 180 W for 10 min, followed by microwave treatment under ice bath conditions (200 W, 83 s) to ensure complete dissolution and homogenization. The resulting mixture was then subjected to rotary evaporation (RE‐52AA, Shanghai Yarong Biochemical Instrument Factory, CN) under reduced pressure at 45°C for 10 min to remove ethanol, forming a gel‐like phase. Subsequently, 30 mL of preheated distilled water (45°C) was added, followed by hydration for 15 min and further sonication at 180 W for 5 min to obtain a stable liposomal suspension for further use.

#### 2.2.2 PCNL‐FD Encapsulation Efficiency Determination

The encapsulation efficiency of PCNL‐FD was determined with slight modifications to the method described by Hao et al. (2024) and Lv et al. (2023). Briefly, 1 mL of PCNL‐FD and blank nanoliposome freeze‐dried powder (BNLF) were placed in centrifuge tubes and centrifuged at 12,000 r/min at 4°C for 30 min. The supernatant was carefully collected and set aside. A 0.5 mL aliquot of the PCNL‐FD supernatant and PCNL‐FD solution was separately mixed with 3 mL of 4% vanillin‐methanol solution and 1.5 mL of concentrated hydrochloric acid. The mixture was vortexed and incubated at 30°C in a dark water bath for 20 min. The absorbance at 500 nm was measured using a multimode microplate reader (H1 Synergy H1, Bio TeK, USA), with BNLF supernatant and BNLF serving as blank controls. The concentration of free PC (C_1_) and total PC (C_2_) was determined using the vanillin‐hydrochloric acid method and a PC standard curve established according to Hao et al. (2024). The encapsulation efficiency of the nanoliposomes was calculated according to Equation (1) below:

(1) $$\text{Encapsulation efficiency}\left(\%\right)=\frac{C_{2}-C_{1}}{C_{2}}\times 100\%$$

In the formula, *C*_1_ represents the concentration of free Proanthocyanidins (μg mL^−1^), and *C*_2_ represents the total procyanidin concentration (μg mL^−1^).

#### 2.2.3 Determination of Particle Size, Polydispersity Index, and Zeta Potential of PCNL‐FD

The particle size distribution, polydispersity index (PDI), and ζ‐potential of PCNL‐FD were measured using a Malvern Particle Sizer (Pro Zetasizer Pro, Malvern, UK) at a testing temperature of (25 ± 0.1)°C.

#### 2.2.4 Procyanidin Nanoliposomal Powder Freeze‐Dried Preservation Agent Screening

To determine the optimal cryoprotectant, different concentrations of trehalose and mannitol were added to the PC‐NL solution. The samples were pre‐frozen at −80°C for 24 h, followed by freeze‐drying using a lyophilizer (GT‐2, Martin Christ Inc., GER). The appearance of the freeze‐dried liposomal samples was observed, and their particle size, PDI, ζ‐potential, and encapsulation efficiency were measured. The best cryoprotectant and its optimal concentration were selected based on a comprehensive evaluation of these parameters.

### 2.3 Characterization of PCNL‐FD

#### 2.3.1 Transmission Electron Microscopy (TEM)

The morphology of PCNL‐FD freeze‐dried powder was observed using a transmission electron microscope (Tecnai 12, FEI Company, NED). The sample was rehydrated with 30 mL of distilled water and diluted tenfold. A 10 μL aliquot of the PCNL‐FD solution was then dropped onto a copper grid, negatively stained with 2.0% phosphotungstic acid, air‐dried naturally, and subsequently examined under the TEM to analyze its structural characteristics.

#### 2.3.2 Fourier Transform Infrared Spectroscopy (FTIR)

For Fourier transform infrared spectroscopy (FTIR) analysis, appropriate amounts of PCNL‐FD, BNLF freeze‐dried powder, and PC were mixed with KBr powder at a mass ratio of 1:100, ground thoroughly, and compressed into pellets. The infrared spectra of the samples were recorded using an FTIR spectrometer (Nicolet iS20, Thermo Fisher Scientific, USA) in the range of 400–4000 cm^−1^. The collected spectra were analyzed to evaluate the structural characteristics and potential interactions between the components.

### 2.4 In Vitro Simulated Digestion of PCNL‐FD

To investigate the in vitro digestion behavior of PCNL‐FD, a simulated digestion model comprising the oral, gastric, and intestinal phases was used, as previously described (Zhang et al. 2019). The simulated digestive fluids, including saliva (SSF), gastric (SGF), intestinal (SIF), duodenal juice, and bile fluid, followed the protocol established by Versantvoort et al. (2005).

#### 2.4.1 Simulated Oral Digestion

The correct amount of PCNL‐FD was rehydrated and combined with SSF in a 1:1 (v/v) ratio before adjusting the pH to 6.8 ± 0.2. Oral digestion was simulated at 100 rpm for 10 min in a 37°C water bath. A PC aqueous solution was used as the control. Samples were collected every 5 min to determine PC concentration, and the proanthocyanidin release rate was calculated according to Formulas (3) and (4).

(3) $$R\left(\%\right)=\left(1-\frac{{\mathrm{EE}}_{t}}{{\mathrm{EE}}_{0}}\right)\times 100\%$$

(4) $$R_{1}\left(\%\right)=\left(1-\frac{A_{t}}{A_{0}}\right)\times 100\%$$

The following formulae are employed in order to calculate the encapsulation efficiency and the PC release rate: EE_t_ is the encapsulation efficiency of the liposomes at different digestion time points. EE_0_ is the initial encapsulation efficiency before digestion. *A*_t_ is the measurement of the absorbance at different digestion time points. *A*_0_ is the initial absorbance before digestion. These parameters are used to evaluate the stability of PCNL‐FD during simulated digestion and to determine the release kinetics of PC in different digestive phases.

#### 2.4.2 Simulates Stomach Digestion

The samples were mixed with an equal volume of simulated gastric fluid, adjusted to pH 2–3, and incubated at 37°C with 100 rpm shaking for 2 h to simulate gastric digestion. A PC aqueous solution was used as the control. Samples were collected every 20 min to measure the PC concentration, and the release rate was calculated using Formulas (3) and (4).

#### 2.4.3 In Vitro Simulated Intestinal Digestion

To simulate intestinal digestion, duodenal juice and bile were mixed at a 2:1 (v/v) ratio to prepare simulated intestinal fluid (SIF). The sample from the gastric digestion stage was then mixed with preheated (37°C) SIF at a 1:1 (v/v) ratio. The pH was adjusted to 7.0, and the mixture was incubated in a shaking water bath at 37°C at 100 rpm for 2 h. A PC aqueous solution (PC solution) was used as the control. Samples were collected every 20 min to determine the PC concentration, and the release rate was calculated using Formulas (3) and (4).

### 2.5 In Vitro Antioxidant Evaluation of PCNL‐FD

#### 2.5.1 Determination of DPPH Free Radical Scavenging Rate

The DPPH radical scavenging activity of PCNL‐FD was determined according to the method described by Jing et al. (2018). The scavenging ability was calculated using Formula (5), and the half‐maximal inhibitory concentration (IC_50_) was determined.

(5) $$\text{DPPH free radical scavenging rate}\left(\%\right)=\left[1-\frac{\left(A_{1}-A_{2}\right)}{A_{3}}\right]\times 100\%$$

In the formula: *A*_1_ represents the absorbance of the sample mixed with the DPPH solution. *A*_2_ represents the absorbance of the sample mixed with anhydrous ethanol. *A*_3_ represents the absorbance of the DPPH solution mixed with anhydrous ethanol.

These values are used to calculate the DPPH radical scavenging activity and determine the half‐maximal inhibitory concentration (IC_50_).

#### 2.5.2 ABTS Radical Scavenging Activity Assay

The ABTS radical scavenging activity of PCNL‐FD was determined according to the method described by Jing et al. (2018). The scavenging ability was calculated using Formula (6), and the half‐maximal inhibitory concentration (IC_50_) was determined.

(6) $$\text{ABTS free radical scavenging rate}\left(\%\right)=\left[1-\frac{\left(A_{4}-A_{5}\right)}{A_{6}}\right]\times 100\%$$

In the formula: *A*_4_ represents the absorbance of the sample mixed with the ABTS solution. *A*_5_ represents the absorbance of the mixture without ABTS. *A*_6_ represents the absorbance of the ABTS solution mixed with ultrapure water.

These values are used to calculate the ABTS radical scavenging activity and determine the half‐maximal inhibitory concentration (IC_50_).

#### 2.5.3 Hydroxyl Radical (OH^·^ ) Scavenging Activity Assay

OH^·^ scavenging activity of PCNL‐FD was determined according to the method described by Zhu et al. (2024). The scavenging ability was calculated using Formula (7), and the half‐maximal inhibitory concentration (IC_50_) was determined.
(7)

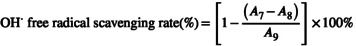




In the formula: *A*_7_ represents the absorbance of the sample mixed with FeSO_4_, salicylic acid‐ethanol solution, and hydrogen peroxide solution. *A*_8_ represents the absorbance of the sample mixed with FeSO_4_ and salicylic acid‐ethanol solution. *A*_9_ represents the absorbance of the mixture of FeSO_4_ solution, salicylic acid‐ethanol solution, and hydrogen peroxide solution.

These values are used to calculate the OH^·^ scavenging activity and determine the half‐maximal inhibitory concentration (IC_50_).

### 2.6 Cell Culture and CD Model Establishment

Caco‐2 cell line has been widely employed in studies on CD‐related damage. Caco‐2 cells (provided by the Kunming Cell Bank, Chinese Academy of Sciences) were cultured in DMEM/F‐12 medium supplemented with 10% (v/v) fetal bovine serum (FBS), 1% (v/v) penicillin, and 1% (v/v) streptomycin. Cells were maintained at 37°C in a humidified atmosphere containing 5% CO_2_. When cell confluency reached approximately 80%, cells were passaged at a 1:3 ratio every 3 days following trypsin digestion.

Caco‐2 cells were randomly divided into five groups: Control group (CK): Untreated normal control. CD model group (CD): Cells treated with 100 μg/mL p31‐43 for 24 h. Blank nanoliposome‐pretreated CD model group (BNLF + CD): Cells pretreated with 20 μg/mL BNLF for 12 h, followed by treatment with 100 μg/mL p31‐43 for 24 h. Proanthocyanidin‐pretreated CD model group (PC + CD): Cells pretreated with 20 μg/mL PC for 12 h, followed by treatment with 100 μg/mL p31‐43 for 24 h. Proanthocyanidin nanoliposome freeze‐dried powder–pretreated CD model group (PCNL‐FD + CD): Cells pretreated with 20 μg/mL PCNL‐FD for 12 h, followed by treatment with 100 μg/mL p31‐43 for 24 h.

The sensitizing peptide p31‐43 was dissolved in PBS to prepare a stock solution. The stock solution was filtered using a 0.22 μM bacterial retention filter and diluted with DMEM/F‐12 medium to final concentrations of 5, 25, 50, 100, 150, and 200 μg/mL. The appropriate concentration was selected to establish the Caco‐2 cell model for celiac disease.

### 2.7 Intervention of PCNL‐FD on Oxidative Stress in a CD Model

#### 2.7.1 Caco‐2 Cell Viability Assay

After completing the respective experimental treatments, Caco‐2 cell viability was assessed. Complete culture medium supplemented with 10% (v/v) CCK‐8 solution was added to the cells, followed by incubation at 37°C for 2 h. Absorbance was recorded at 450 nm using a multimode microplate reader (H1 Synergy H1, Bio TeK, USA).

#### 2.7.2 Oxidative Stress Measurement

Caco‐2 cells were incubated in 6‐well plates for 24 h, after which they were collected to assess oxidative stress levels. The levels of SOD, GSH, GSSG, and MDA were used as oxidative stress indicators and were measured according to the respective manufacturers' instructions using microplate detection systems. The intracellular ROS levels were assessed using the DCFH‐DA probe method. Cells were observed under a laser confocal microscope, and ImageJ software was used to analyze the mean fluorescence intensity, which was used to quantify ROS levels.

#### 2.7.3 Quantification of Inflammatory Mediators

Caco‐2 cells were incubated in 6‐well plates for 24 h, after which the cell culture supernatant was collected. The levels of inflammatory mediators, including IL‐4, IL‐10, TNF‐α, and IL‐6, were measured using enzyme‐linked immunosorbent assay (ELISA) kits.

#### 2.7.4 Western Blot Analysis of Keap1/Nrf2 and NQO‐1/HO‐1 Signaling Pathways in a CD Model With PCNL‐FD Intervention

Total protein was extracted from Caco‐2 cells using RIPA buffer containing protease inhibitors. After quantification, protein samples were separated by 10% SDS‐PAGE and transferred to PVDF membranes. The membranes were blocked with 5% BSA or skimmed milk for 1.5 h at room temperature, followed by overnight incubation at 4°C with primary antibodies against Keap1, Nrf2, NQO‐1, and HO‐1. After washing six times with TBST (6 min each), the membranes were incubated with HRP‐conjugated secondary antibodies for 1 h at room temperature. Following another six TBST washes, protein bands were detected using an ECL kit (Wang, Cui, Xu, et al. 2022).

### 2.8 Statistical Analysis

Experimental data were compiled in Excel 2021, followed by statistical analysis using SPSS 26. GraphPad Prism 8 and Design‐Expert 13 were used for data visualization and experimental design. Western blot and ROS fluorescence results were quantified using ImageJ software. Results are expressed as mean ± standard deviation (SD). Statistical significance was determined by one‐way ANOVA followed by Tukey's test for multiple comparisons, with *p* < 0.05 considered statistically significant. All experiments were performed independently at least three times (*n* ≥ 3).

## 3 Results and Discussion

### 3.1 Effects of Lyophilization Protectants on Procyanidin Nanoliposomes

**FIGURE 1:**
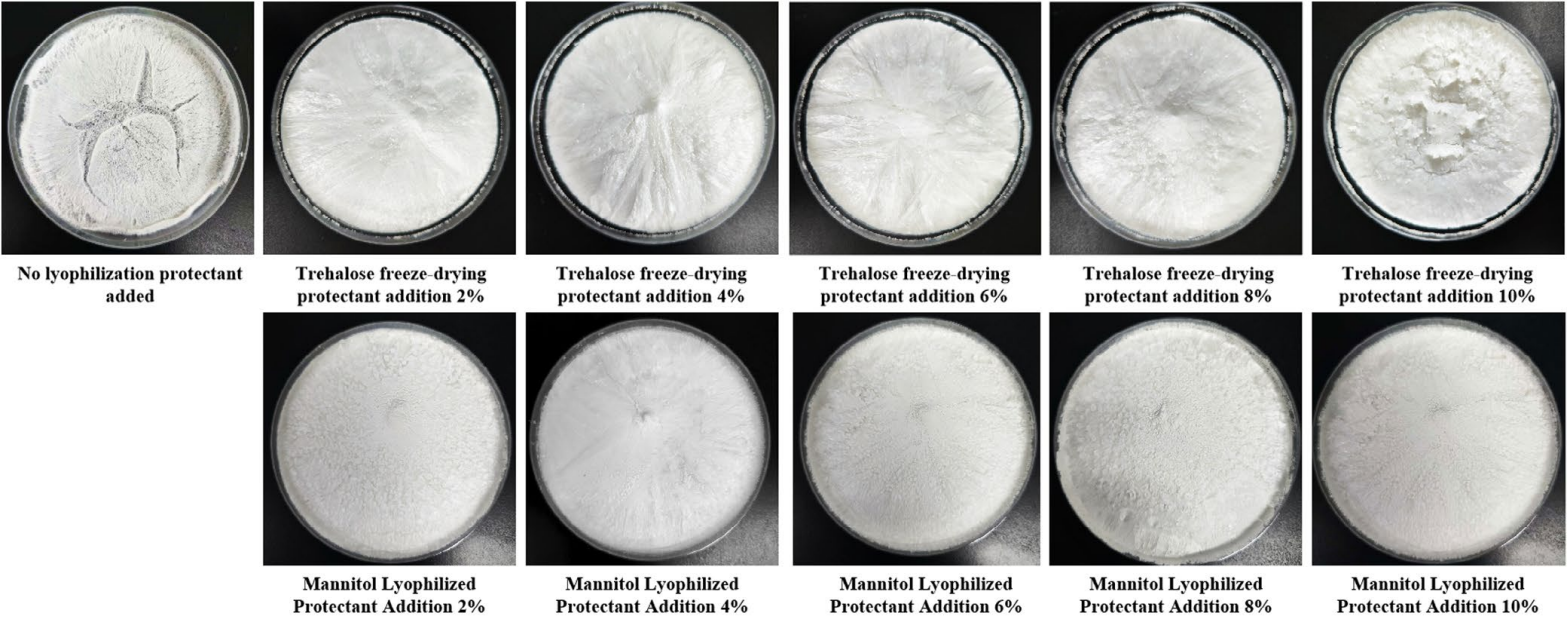
Effect of lyophilization protectant agent on the appearance of PC‐NL lyophilized powder.

A smaller particle size and a higher absolute ζ‐potential are critical for the stability of nanoliposomes as nano‐delivery systems (Toro‐Uribe et al. 2019). As shown in Table 1 and Figure 1, the polydispersity index (PDI) reflects the uniformity of the particle size distribution, with lower PDI values indicating a more homogeneous size distribution (Santos et al. 2019). Before freeze‐drying, the PCNL‐FD solution exhibited a particle size of 182.96 nm, a PDI of 0.247, a ζ‐potential of −18.43 mV, and an encapsulation efficiency of 94.84%.

**TABLE 1:** Effect of lyophilization protectant agent on particle size, PDI, ζ potential and encapsulation efficiency of PC‐NL.

Lyophilization protectant type	Additive amount (%)	Particle size/nm	PDI	ζ‐potential/mV	Encapsulation rate (100%)
Trehalose	0	274.985 ± 2.168^a^	0.390 ± 0.003^a^	−9.600 ± 0.163^a^	39.619 ± 8.402^d^
	2	190.123 ± 1.413^d^	0.254 ± 0.004^b^	−16.000 ± 0.668^c^	84.322 ± 2.491^b^
	4	182.713 ± 0.967^e^	0.225 ± 0.008^d^	−18.700 ± 0.648^e^	91.917 ± 2.775^a^
	6	184.380 ± 0.738^e^	0.228 ± 0.001^d^	−17.300 ± 1.329^d^	92.694 ± 5.038^a^
	8	198.218 ± 1.029^c^	0.244 ± 0.004^c^	−11.425 ± 0.866^b^	72.745 ± 4.726^c^
	10	211.340 ± 1.596^b^	0.247 ± 0.006^ab^	−10.600 ± 0.909^ab^	74.481 ± 6.640^c^
Mannitol	0	274.985 ± 2.168^f^	0.390 ± 0.003^b^	−9.600 ± 0.163^a^	39.619 ± 8.402^d^
	2	346.255 ± 6.934^e^	0.307 ± 0.004^d^	−15.925 ± 0.126^d^	89.482 ± 1.217^a^
	4	324.358 ± 1.149^d^	0.269 ± 0.009^e^	−18.100 ± 0.163^e^	92.918 ± 0.879^a^
	6	363.803 ± 1.665^c^	0.303 ± 0.002^d^	−16.175 ± 0.096^d^	79.915 ± 2.635^b^
	8	379.013 ± 0.750^b^	0.355 ± 0.007^c^	−12.525 ± 0.591^c^	79.583 ± 2.233^b^
	10	404.345 ± 3.351^a^	0.465 ± 0.022^a^	−11.450 ± 0.866^b^	70.284 ± 6.065^c^

*Note:* Different superscript letters in the same column indicate significant differences (*p* < 0.05).

As shown in Table 1 and Figure 1, the freeze‐dried PCNL‐FD sample without lyophilization protectant appeared light yellow, with noticeable shrinkage and cracks. After rehydration, the particle size increased to 274.985 nm, the PDI to 0.390, and the ζ‐potential decreased to −9.600 mV. The encapsulation efficiency dropped significantly to 39.619%, indicating a marked reduction in stability and encapsulation efficiency compared to the pre‐lyophilization sample (*p* < 0.05). When trehalose and mannitol were used as cryoprotectants, the freeze‐dried PCNL‐FD samples exhibited improved structural integrity and a milky‐white appearance, preventing severe collapse and cracking. However, as the concentration of trehalose increased, the surface of the freeze‐dried samples transitioned from smooth and uniform to progressively collapsed and wrinkled. In contrast, for mannitol as a cryoprotectant, the sample surface remained relatively smooth at a concentration of 4%, but when the concentration was either lower or higher than 4%, the number of surface bubbles and depressions increased.

With increasing concentrations of trehalose and mannitol, the particle size, PDI, and ζ‐potential initially decreased and then increased, while the encapsulation efficiency followed a similar trend. This phenomenon may be attributed to fusion or aggregation between nanoliposome particles due to water loss during freeze‐drying (Chen et al. 2010). Upon rehydration, the added water could have induced bilayer expansion, leading to an increase in particle size (Stark et al. 2010). Additionally, during freezing and drying, cryoprotectants exerted various stresses on the liposomal suspension to maintain its structural integrity, which could also have contributed to particle aggregation and increased size (Wu et al. 2019). An increase in particle size could also elevate liposome leakage rates, resulting in content loss and ultimately affecting encapsulation efficiency (Jarzynska et al. 2024).

The particle size of nanocarriers plays a crucial role in their accumulation and penetration at diseased sites, with the enhanced permeability and retention (EPR) effect typically being effective for nanoparticles within the size range of 30–200 nm (Zhao et al. 2019). When the concentration of the cryoprotectant was set at 4%, the encapsulation efficiency of both trehalose and mannitol exceeded 91%. However, the particle size of trehalose‐protected PCNL‐FD was significantly smaller at 182.713 ± 0.967 nm, compared to 324.358 ± 1.149 nm for mannitol‐protected PCNL‐FD. Considering both encapsulation efficiency and particle size, 4% trehalose was ultimately selected as the cryoprotectant for PCNL‐FD freeze‐drying.

### 3.2 Characterization of PCNL‐FD

#### 3.2.1 Transmission Electron Microscopy (TEM)

**FIGURE 2:**
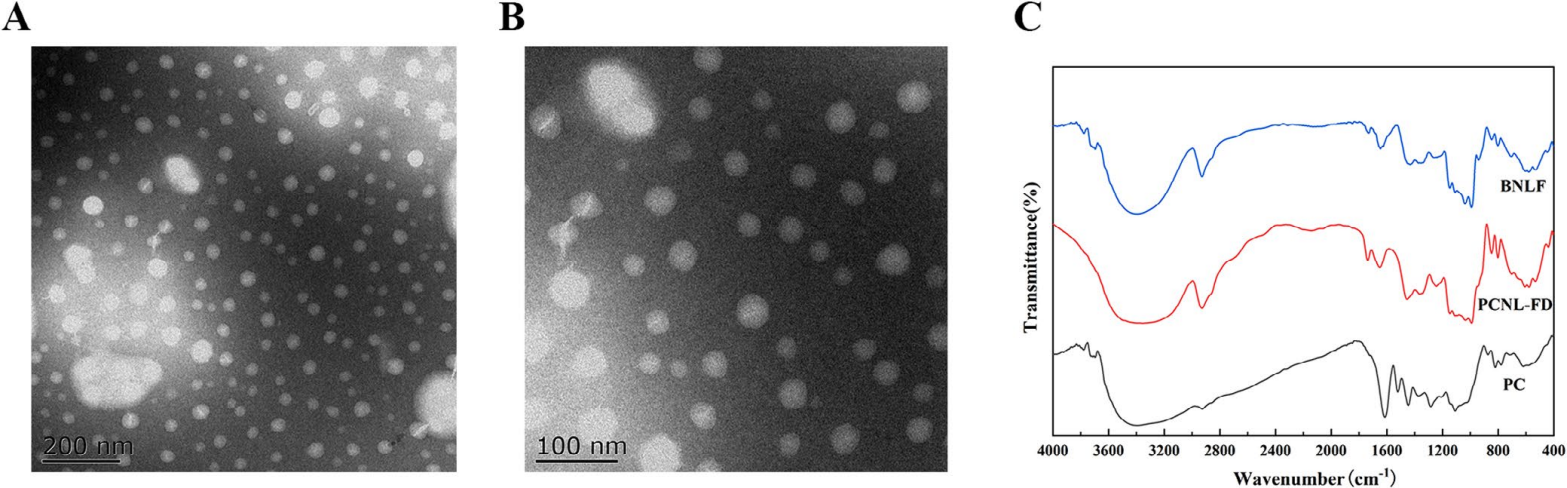
Characterization of PCNL‐FD. (A, B) Transmission electron microscopy (TEM) image of PCNL‐FD. (C) FTIR spectrum of PCNL‐FD.

**FIGURE 3:**
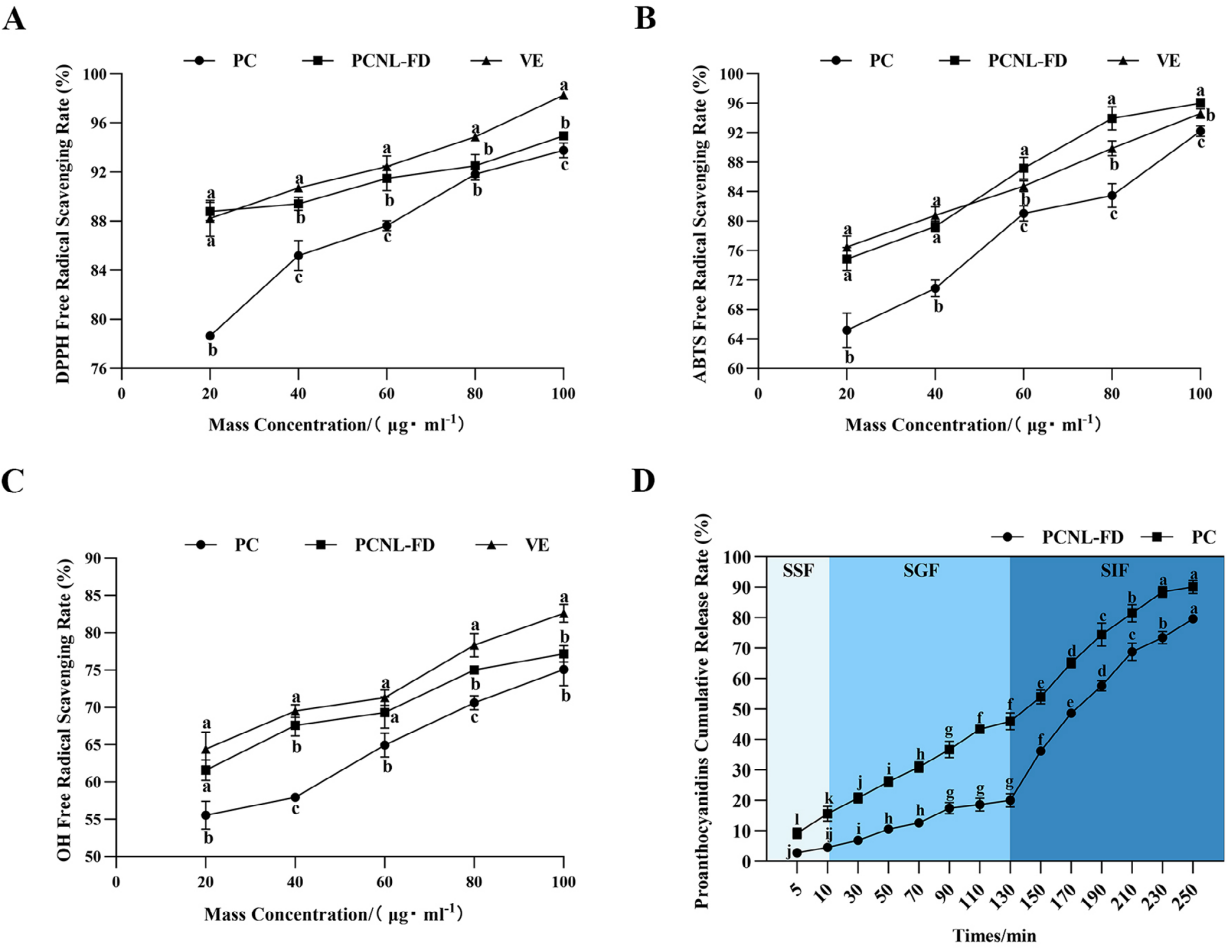
In Vitro antioxidant evaluation of PCNL‐FD with in vitro simulated digestion. (A) The effect of PC, PCNL‐FD, and VE on DPPH free radical scavenging rate. (B) The effect of PC, PCNL‐FD, and VE on ABTS radical scavenging rate. (C) The effect of PC, PCNL‐FD, and VE on OH radical scavenging rate. (D) In vitro simulated digestion of PCNL‐FD. Different letters indicate significant differences (*p* < 0.05).

The shape and size of nanoparticles significantly influence their solubility and fluidity. As shown in Figure 2A,B, the microstructural morphology of PCNL‐FD was observed using TEM, with detailed images presented in Figure 3 The results indicate that PCNL‐FD exhibits an ellipsoidal, smooth, and nearly uniform morphology with a narrow particle size distribution. The aggregation of nanoliposomes was observed, which is attributed to the fusion and coalescence of lipid bilayers during the preparation process.

#### 3.2.2 Fourier Transform Infrared Spectroscopy (FTIR)

As shown in Figure 2C, a comparison of the FTIR spectra of BNLF and PCNL‐FD confirmed the presence of several characteristic absorption peaks of liposomes: The broad peak at 3300 cm^−1^ corresponds to the presence of O‐H functional groups. The peaks at 2927 and 2867 cm^−1^ are associated with the asymmetric and symmetric stretching vibrations of C‐H bonds, respectively (Jordanov et al. 2003). The peaks at 1736, 1145, and 1039 cm^−1^ correspond to the stretching vibrations of C=O, PO_2_, and P‐O‐C groups in the phospholipid structure (Pilch and Musiał 2018). In the FTIR spectrum of PC, a broad absorption band peaking at 3397 cm^−1^ is assigned to hydrogen bonding between phenolic hydroxyl groups (Biao et al. 2018). The absorption bands at 1613 and 1107 cm^−1^ are attributed to characteristic functional groups of polyflavonoids (Chen et al. 2024; Ku and Mun 2007). Notably, the absorption peak around 1285 cm^−1^ indicates the presence of flavonoid‐based tannins (Ding et al. 2021). The FTIR spectrum of PCNL‐FD closely resembles that of BNLF, with minimal characteristic peaks of PC, suggesting that PC has been successfully embedded within the phospholipid bilayer (Luo et al. 2020).

### 3.3 In Vitro Antioxidant Evaluation and In Vitro Simulated Digestion of PCNL‐FD

#### 3.3.1 In Vitro Antioxidant Evaluation

DPPH is a stable free radical that decreases in absorbance when neutralized by antioxidants through hydrogen atom donation, indicating the radical‐scavenging ability of antioxidants (Kim and Lee 2009). The ABTS assay reflects the ability of antioxidants to scavenge ABTS radicals via electron or hydrogen transfer and is commonly used to assess the primary radical‐scavenging activity of antioxidants (Limsuwanmanee et al. 2014). Among various reactive oxygen species, hydroxyl radicals (OH^·^) are considered the most destructive. These highly reactive free radicals can severely damage cellular biomolecules and cause lipid peroxidation in liposomal membranes, leading to membrane disruption and potential content leakage (Reis and Spickett 2012).

As shown in Figure 3A–C, the DPPH, ABTS, and OH radical scavenging rates increased with increasing concentrations of PC, PCNL‐FD, and VE (vitamin E, used as a reference antioxidant). The calculated IC_50_ values for DPPH radical scavenging were 3.779, 0.1515, and 1.358 μg/mL for PC, PCNL‐FD, and VE, respectively. The IC_50_ values for ABTS radical scavenging were 10.85, 7.327, and 4.634 μg/mL, while the IC_50_ values for OH radical scavenging were 15.86, 7.340, and 7.249 μg/mL, respectively.

PCNL‐FD exhibited lower IC_50_ values for DPPH, ABTS, and OH radicals compared to PC, indicating enhanced radical‐scavenging efficiency. Additionally, the scavenging rates observed in the figures show that PCNL‐FD demonstrated significantly higher antioxidant activity than PC across all three assays. This suggests that liposomal encapsulation protects PC from external environmental degradation, thereby enhancing its antioxidant efficacy. These findings are consistent with previous research (Luo et al. 2020; Sun et al. 2024).

#### 3.3.2 In Vitro Simulated Digestion

The results of the simulated oral digestion phase are shown in Figure 3D SSF digestion process. Within the first 10 min of oral digestion, the cumulative release rate of PC from PCNL‐FD remained relatively unchanged, indicating that the liposomal structure remained largely intact in the oral cavity and was not significantly digested. In contrast, the release rate of free PC increased significantly, likely due to the absence of a phospholipid bilayer, making it more susceptible to enzymatic degradation by salivary amylase (Ismail et al. 2021).

A similar trend was observed during the SGF. The leakage rate of PCNL‐FD reached 20.038% ± 2.168%, representing only a 15% increase compared to the SSF phase. This relatively low leakage is attributed to the well‐organized lipid bilayer structure of liposomes, where cholesterol forms hydrogen bonds with phospholipids, increasing membrane rigidity and enhancing the structural stability of liposomes against gastric stress (Liu et al. 2019). Additionally, gastric lipase lacks activity against phospholipids (Carriere et al. 1991), preventing significant phospholipid hydrolysis in the stomach. Conversely, free PC, which lacks phospholipid encapsulation, underwent rapid degradation in the gastric environment, leading to a significant increase in its release rate.

During the SIF, a significant increase in the cumulative release rate of PC from PCNL‐FD was observed, indicating that the phospholipid bilayer of the liposomes was extensively disrupted. This phenomenon is primarily due to pancreatic enzymes, including pancreatic lipase, phospholipase A_2_, and cholesterol esterase, which catalyze phospholipid hydrolysis, ultimately disrupting the lipid bilayer and facilitating content release (Liu et al. 2020). Additionally, bile salts present in the intestinal fluid can adsorb onto or insert into the phospholipid bilayer, increasing membrane fluidity and enhancing the interaction between pancreatic enzymes and phospholipids, further compromising bilayer integrity (Liu et al. 2020, 2018).

Notably, the release rate of free PC remained significantly higher than that of PCNL‐FD at this stage, suggesting that phospholipid encapsulation improves the bioavailability of polyphenolic compounds and enables controlled release over time. This finding aligns with the results reported by Lv et al. (2023), which demonstrated that encapsulation enhances the gastrointestinal stability of polyphenolic compounds and facilitates controlled release at the intended target site.

### 3.4 Establishment of the In Vitro Celiac Disease Cell Model and PCNL‐FD Concentration Screening

#### 3.4.1 Establishment of the In Vitro Celiac Disease Cell Model

**FIGURE 4:**
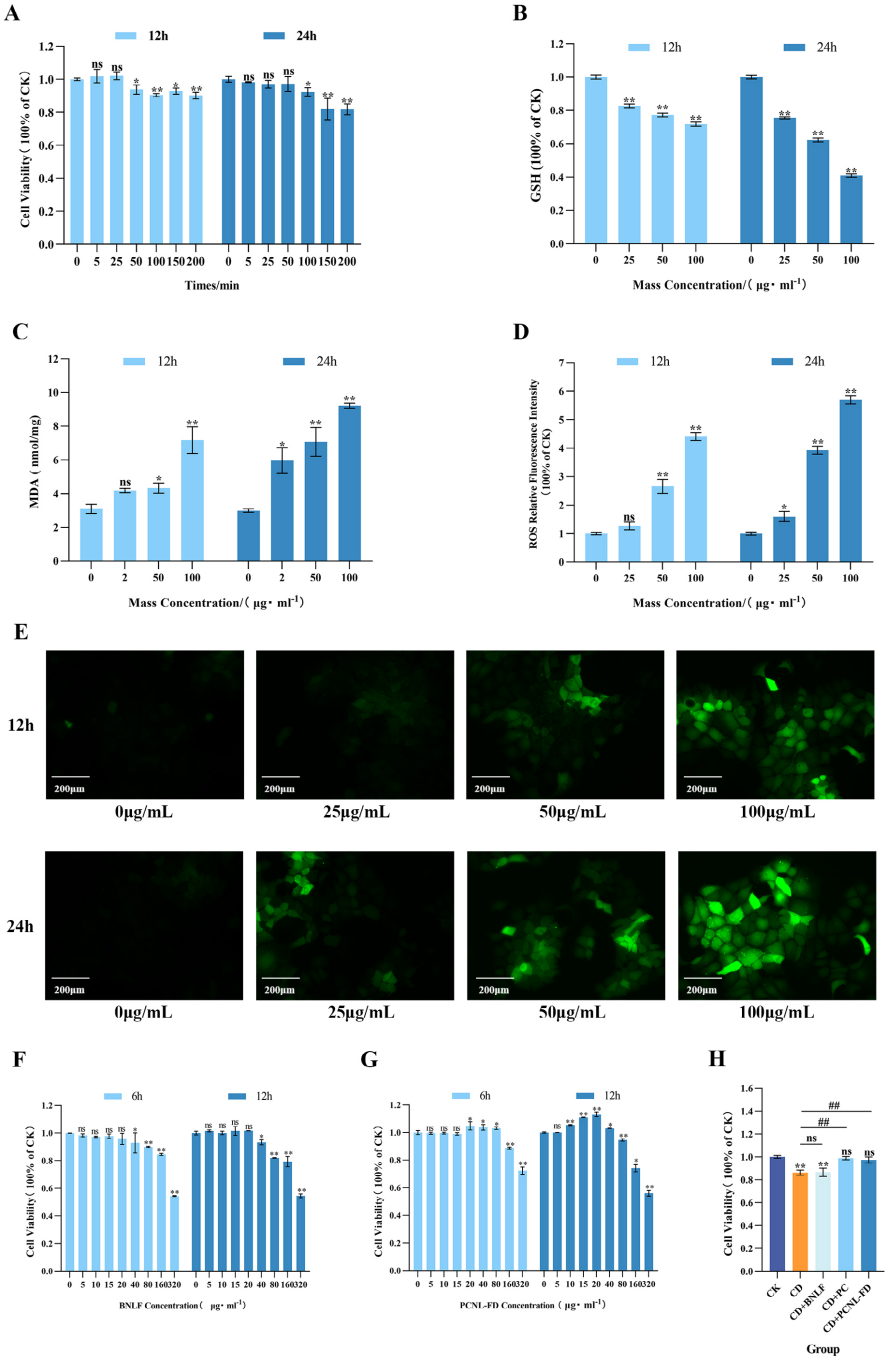
Establishment of the in vitro celiac disease cell model and PCNL‐FD concentration screening. (A) The effect of gliadin peptide p31‐43 stimulation on the viability of Caco‐2 cells. (B) The impact of gliadin peptide p31‐43 on intracellular GSH levels in Caco‐2 cells. (C) The impact of gliadin peptide p31‐43 on intracellular MDA levels in Caco‐2 cells. (D) Quantitative analysis of ROS fluorescence expression in Caco‐2 cells induced by gliadin peptide p31‐43. (E) Fluorescence expression of ROS in Caco‐2 cells induced by gliadin peptide p31‐43. (F) The effect of BNLF on the viability changes of Caco‐2 cells. (G) The effect of PCNL‐FD on the viability changes of Caco‐2 cells. (H) The protective effect of PC, BNLF, and PCNL‐FD on the viability of model cells. ** indicates a statistically significant and highly significant difference compared to the CK group at *p < 0.05; **p < 0.001; ^##^ indicates a statistically significant and highly significant difference between two groups at ^#^ *p* < 0.05; ^##^ *p* < 0.001, the same as below.

The concentration and exposure time of the gluten‐derived sensitizing peptide p31‐43 in the Caco‐2 oxidative damage model were determined using the CCK‐8 assay. As shown in Figure 4A, compared with the control group, after 12 and 24 h of treatment, Caco‐2 cell viability remained above 90% for peptide concentrations ranging from 5 to 100 μg/mL. Therefore, 25, 50, and 100 μg/mL peptide concentrations were selected for subsequent GSH, ROS, and MDA measurements.

GSH is an important non‐enzymatic antioxidant that plays a crucial role in cellular redox balance (Allen and Bradley 2011). As shown in Figure 4B, at both 12 and 24 h, low, medium, and high concentrations of p31‐43 significantly reduced intracellular GSH levels (*p* < 0.001). When the exposure time reached 24 h and the peptide concentration was 100 μg/mL, GSH levels decreased to their lowest point, indicating that this concentration‐time combination induced the most pronounced oxidative stress. This finding contrasts with previous studies showing the protective effects of antioxidant peptides on Caco‐2 cells (Li, Li, et al. 2024).

ROS and MDA are key biomarkers of oxidative stress. ROS comprises highly reactive oxygen species, including superoxide anions, hydrogen peroxide, and hydroxyl radicals (Jomova et al. 2024). MDA is a lipid peroxidation product, and its elevated levels are generally associated with oxidative stress and cellular damage (Martins et al. 2022). As shown in Figure 4C–E, after 24 h of exposure to 100 μg/mL p31‐43, MDA levels peaked, and ROS levels significantly increased to their highest values (*p* < 0.001), indicating that this concentration–time combination induced the most severe oxidative stress. This observation aligns with the findings of Nanayakkara et al. (2018), who reported that 100 μg/mL p31‐43 treatment for 24 h led to a significant increase in ROS levels and maximum MDA accumulation.

By integrating the results for GSH, MDA, and ROS, it was confirmed that 100 μg/mL p31‐43 exposure for 24 h induced the most severe oxidative damage in Caco‐2 cells. Therefore, this concentration and time point were selected as the standard conditions for p31‐43‐induced oxidative stress in subsequent experiments.

#### 3.4.2 PCNL‐FD Concentration Screening

As shown in Figure 4F, when the BNLF concentration was 20 μg/mL, the cell viability at 6 h was 95%, which was lower than the 100% viability at 12 h. Therefore, 12 h was selected as the optimal pre‐treatment time. Similarly, as shown in Figure 4G, when the PCNL‐FD concentration was 20 μg/mL, the cell viability at 6 h was 105%, which was lower than the 113% viability at 12 h. Hence, 12 h was chosen as the pre‐treatment duration for PCNL‐FD as well. As shown in Figure 4H, compared with the CD model group, PCNL‐FD significantly increased cell viability (*p* < 0.001), while BNLF had no significant effect on cell viability. Consequently, BNLF was excluded from further experiments. These results indicate that PCNL‐FD at a concentration of 20 μg/mL effectively mitigated the cytotoxic effects of the sensitizing peptide p31‐43 (100 μg/mL) in the CD model to some extent.

### 3.5 Intervention of PCNL‐FD on the Oxidative Stress Level in CD Model

#### 3.5.1 Effects of PCNL‐FD on Oxidative Stress in CD Model

**FIGURE 5:**
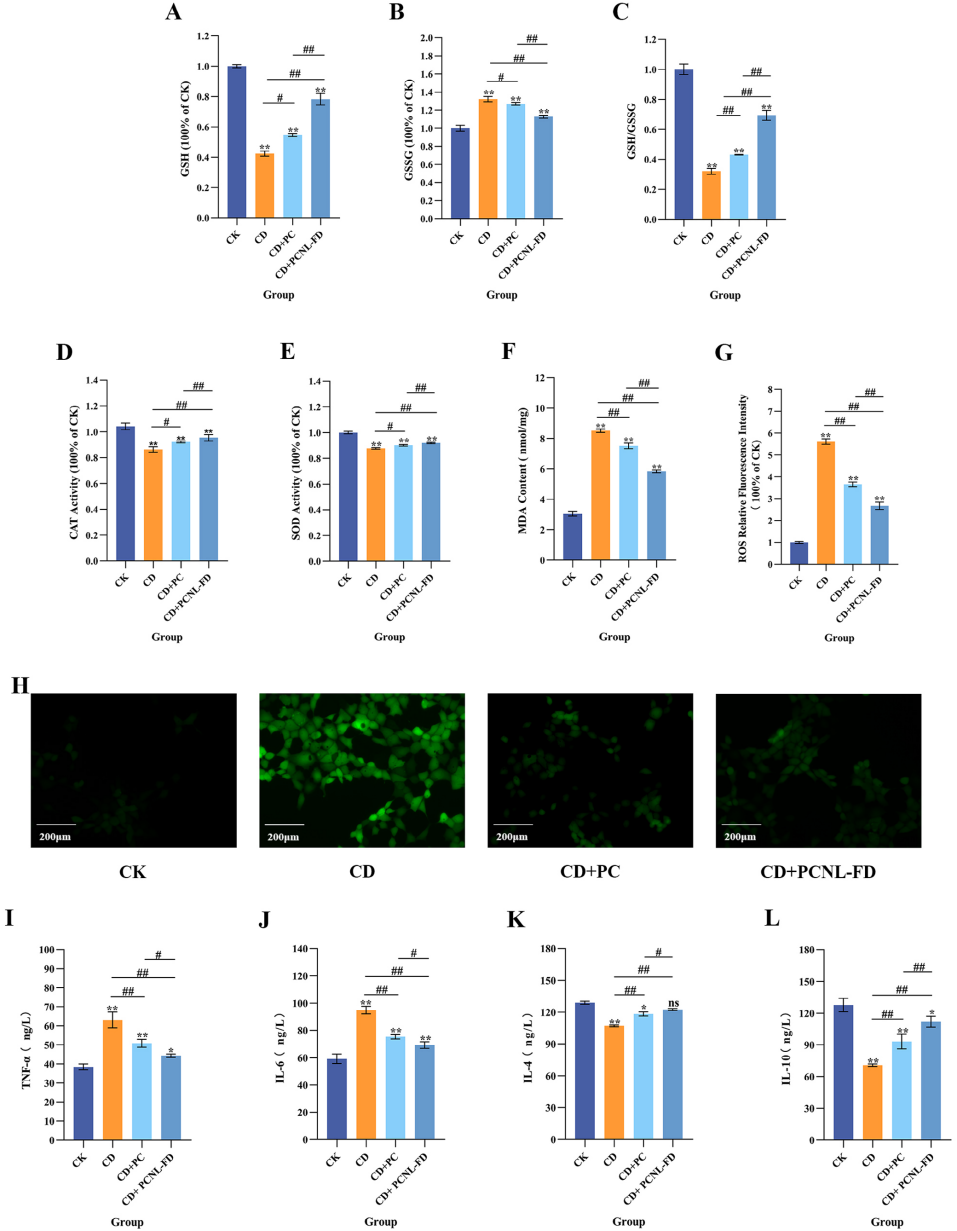
The effect of PCNL‐FD on oxidative stress in the CD cell model. (A) The effect of PCNL‐FD treatment on intracellular GSH levels. (B) The effect of PCNL‐FD treatment on intracellular GSSG levels. (C) The effect of PCNL‐FD treatment on the intracellular GSH/GSSG ratio. (D) The effect of PCNL‐FD treatment on intracellular CAT activity. (E) The effect of PCNL‐FD treatment on intracellular SOD activity. (F) The effect of PCNL‐FD treatment on intracellular MDA levels. (G) Quantitative analysis of ROS fluorescence expression in cells. (H) Intracellular ROS fluorescence expression levels. (I) The level of TNF‐α in Caco‐2 cells. (J) The level of IL‐6 in Caco‐2 cells. (K) The level of IL‐4 in Caco‐2 cells. (L) The level of IL‐10 in Caco‐2 cells.

GSSG is the oxidized form of GSH, and its concentration increases under oxidative stress, leading to a reduction in the GSH/GSSG ratio (Franco and Cidlowski 2009). Figure 5A shows that p31‐43 treatment caused a significant decrease in intracellular GSH levels (*p* < 0.001), whereas PCNL‐FD pre‐treatment significantly increased GSH levels in the CD model group (*p* < 0.001). Figure 5B indicates that PCNL‐FD pre‐treatment significantly reduced GSSG levels in the CD model group (*p* < 0.001). Figure 5C shows that the GSH/GSSG ratio significantly increased (*p* < 0.001) after PCNL‐FD pre‐treatment, demonstrating its ability to alleviate p31‐43‐induced oxidative stress, reduce GSSG levels, and enhance intracellular GSH content.

CAT and SOD are key antioxidant enzymes that play crucial roles in cellular defense against oxidative damage by efficiently scavenging ROS, thereby mitigating oxidative stress‐induced cellular injury (Cui et al. 2023). Figure 5D,E demonstrates that CAT and SOD activity were significantly increased (*p* < 0.05) in the CD model group pre‐treated with PCNL‐FD compared to the untreated CD group. These results suggest that PCNL‐FD pre‐treatment enhances cellular antioxidant enzyme activity, thereby improving the cells' defense mechanisms against oxidative stress. Figure 5F indicates that PCNL‐FD pre‐treatment effectively reduced MDA levels, mitigating p31‐43‐induced lipid peroxidation, although MDA levels did not fully return to normal. This finding aligns with a study by Qu et al. (2019), which reported that α‐tocopherol liposomes encapsulated in chitosan hydrogels significantly reduced ROS levels in cardiomyocytes. Figures 5G and 5H illustrate that compared to the CK group, the CD group exhibited a significant increase in ROS fluorescence intensity, indicating elevated ROS levels. However, PCNL‐FD pre‐treatment significantly decreased ROS fluorescence intensity (*p* < 0.001), suggesting that PCNL‐FD effectively reduced intracellular ROS accumulation. This phenomenon may be attributed to the activation of intracellular antioxidant defense mechanisms, such as upregulating the activity of key antioxidant enzymes, including SOD and CAT.

Furthermore, as observed in Figure 5A–H, the protective effect of PCNL‐FD pre‐treatment was significantly greater than that of free PC (*p* < 0.001). This finding suggests that nanoliposomal encapsulation enhances the antioxidant capacity of PC, allowing it to exert stronger intracellular antioxidant activity. Similarly, Liu, Zhou, et al. (2025) reported that quercetin encapsulated in liposomes exhibited superior antioxidant activity compared to free quercetin. This enhanced effect was achieved by increasing intracellular SOD and CAT activity while reducing ROS and MDA levels, thereby exerting a more potent antioxidant effect.

#### 3.5.2 Effects of PCNL‐FD on Inflammatory Factors in CD Model

During the progression of CD, the increase in ROS and MDA levels often triggers the secretion of pro‐inflammatory cytokines such as TNF‐α and IL‐6 (Moretti et al. 2018). TNF‐α, primarily secreted by intestinal epithelial cells and immune cells in the lamina propria, including macrophages and lymphocytes, activates signaling pathways such as NF‐κB, promoting the release of additional inflammatory cytokines and exacerbating mucosal inflammation in the intestine (Khan et al. 2016; Marafini et al. 2015). IL‐6 plays a synergistic role in inflammation by stimulating the synthesis of acute‐phase proteins and enhancing immune cell activation, thereby further aggravating intestinal inflammation (Popko et al. 2010). As shown in Figure 5I,J, compared to the CK group, the CD group exhibited a significant increase in TNF‐α and IL‐6 levels (*p* < 0.001), while pre‐treatment with PC and PCNL‐FD significantly reduced their levels (*p* < 0.001), indicating that PCNL‐FD effectively suppressed the inflammatory response. In contrast, IL‐4 and IL‐10, which play key roles in regulating immune responses in CD, were significantly decreased in the CD group compared to the CK group (*p* < 0.001) (Figure 5K,L). IL‐4, primarily secreted by Th2 cells, inhibits Th1‐mediated inflammatory responses and reduces intestinal damage (Forsberg et al. 2002), while IL‐10 is an important anti‐inflammatory cytokine that suppresses the production of pro‐inflammatory cytokines such as IFN‐γ and TNF‐α, thereby alleviating intestinal inflammation (Passerini et al. 2024). Following PC and PCNL‐FD pre‐treatment, IL‐4 and IL‐10 levels were significantly increased (*p* < 0.001), demonstrating that PCNL‐FD effectively modulated immune responses by enhancing anti‐inflammatory cytokine levels. In summary, PCNL‐FD exerted a dual regulatory effect on cytokine secretion in the CD model by suppressing excessive secretion of pro‐inflammatory cytokines (TNF‐α and IL‐6), thereby reducing intestinal inflammation, while simultaneously promoting the secretion of anti‐inflammatory cytokines (IL‐4 and IL‐10), enhancing the body's anti‐inflammatory response, and aiding in the restoration of intestinal homeostasis. Notably, PCNL‐FD exhibited a superior anti‐inflammatory effect compared to PC (*p* < 0.001), suggesting that liposomal encapsulation effectively protected PC from degradation in the biological environment, thereby significantly enhancing its bioavailability and targeting ability.

### 3.6 Effects of PCNL‐FD on the Keap1/Nrf2 and NQO‐1/HO‐1 Signaling Pathways in a CD Model

**FIGURE 6:**
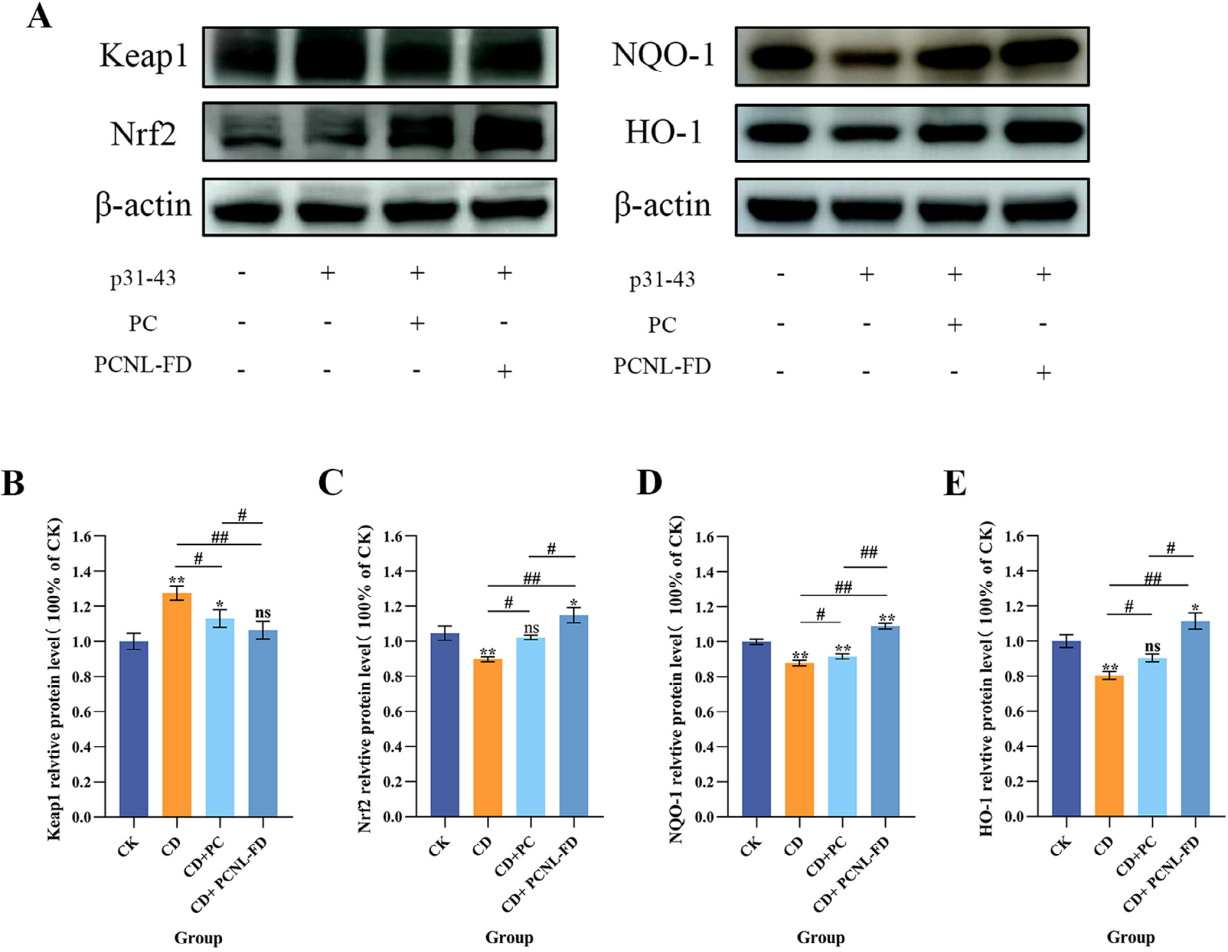
Effect of PCNL‐FD on the protein expression of Keap1/Nrf2 and NQO‐1/HO‐1 in CD cell model. (A) Intracellular Keap1, Nrf2, NQO‐1, and HO‐1 protein expression levels. (B) Relative protein expression levels of Keap1. (C) Relative protein expression levels of Nrf2. (D) Relative protein expression levels of NQO‐1. (E) Relative protein expression levels of HO‐1.

The Keap1/Nrf2 signaling pathway and its downstream targets NQO‐1 and HO‐1 play crucial roles in cellular antioxidant defense. As shown in Figure 6, the activation of signaling pathways in the PCNL‐FD pre‐treated CD model group exhibited distinct differences compared to the untreated CD group. In p31‐43‐treated CD cells, the expression of Keap1 protein was significantly increased compared to the CK group (*p* < 0.001), while Nrf2, NQO‐1, and HO‐1 protein levels were significantly reduced (*p* < 0.001). However, in the PCNL‐FD pre‐treated CD model group, Keap1 expression was significantly reduced (*p* < 0.001), while Nrf2, NQO‐1, and HO‐1 expression levels were significantly increased (*p* < 0.001) compared to the untreated CD group.

As previously mentioned, gliadin peptide p31‐43 promotes adaptive immune responses in CD primarily by increasing ROS levels (Wang, Cui, Xu, et al. 2022). ROS interacts with Keap1, leading to conformational changes in Keap1 (Zhang et al. 2016). Theoretically, this interaction should cause Keap1 to dissociate from Nrf2, allowing Nrf2 to enter the nucleus and activate antioxidant genes. However, sustained oxidative stress may push the cells into a state of “oxidative stress imbalance,” leading to increased Keap1 expression, which further suppresses Nrf2 activity as a compensatory mechanism to maintain intracellular redox balance (Wang, Cui, Li, et al. 2022). These findings demonstrate that in the CD model group, Keap1 expression was significantly upregulated (*p* < 0.001), accompanied by a significant downregulation of Nrf2 expression.

Additionally, previous studies have indicated that Keap1 can interact with other proteins beyond Nrf2, such as p62 and phosphoglycerate mutase 5 (PGAM5) (Tonelli et al. 2018). Similarly, Nrf2 activity can be regulated by phosphorylation, involving pathways such as protein kinase C (PKC) (Tonelli et al. 2018) and mitogen‐activated protein kinases (MAPKs) (Sun et al. 2009). Furthermore, cell apoptosis may also contribute to Nrf2 degradation (He et al. 2019).

In the PCNL‐FD pre‐treated CD model group, both Keap1 and Nrf2 protein expression levels were significantly upregulated (*p* < 0.001), indicating that PCNL‐FD may enhance cellular antioxidant capacity by modulating the Keap1/Nrf2 signaling pathway. Specifically, the reduction in Keap1 levels suggests an adaptive regulatory response to oxidative stress, while the increase in Nrf2 expression indicates that its downstream antioxidant genes, such as NQO‐1 and HO‐1, were activated (Fang et al. 2021; Li et al. 2014). As downstream target genes of the Nrf2 signaling pathway, NQO‐1 and HO‐1 exhibited significantly increased expression levels (*p* < 0.001) in the PCNL‐FD pre‐treated CD model group, suggesting that PCNL‐FD enhances antioxidant gene expression, thereby mitigating oxidative stress‐induced cellular damage.

Overall, both PCNL‐FD and PC contributed to restoring Keap1/Nrf2 and NQO‐1/HO‐1 protein expression to homeostatic levels, with PCNL‐FD demonstrating superior antioxidant effects compared to PC (*p* < 0.05). This enhanced efficacy may be attributed to the liposomal structure's similarity to the cell membrane, which facilitates improved cellular uptake and enhances its antioxidant effects (Pan et al. 2024). Similar findings were reported by Liu, Zhou, et al. (2025), who found that liposomal encapsulation of quercetin effectively regulated oxidative stress‐related genes such as HO‐1, Keap1, and Nrf2, activated downstream antioxidant enzymes, and upregulated NQO‐1 and Keap1 expression, thereby alleviating H_2_O_2_‐induced oxidative damage.

## 4 Strengths and Limitations

PCNL‐FD, prepared using trehalose as a lyoprotectant, exhibited excellent physicochemical properties and sustained‐release characteristics, offering a novel approach for CD‐intervention foods. For the first time, we explored PCNL‐FD's protective effects against p31‐43‐induced celiac disease in a cellular model. The results demonstrated that PCNL‐FD exerts antioxidant and anti‐inflammatory effects via regulating the Keap1/Nrf2 pathway and promoting the expression of downstream antioxidant genes (NQO‐1 and HO‐1). These findings highlight its potential as a novel strategy for mitigating oxidative damage in CD.

Yet CD is a common, chronic, immune‐mediated small bowel enteropathy resulting from gluten exposure in genetically susceptible individuals. So, the targeting ability and bioavailability of PC‐NL still require further improvement. The current study was limited to in vitro cellular models; a systematic evaluation of the pharmacokinetic characteristics of PC‐NL, including its absorption, distribution, metabolism, and excretion (ADME) in vivo, remains uncharacterized. These limitations hinder a comprehensive understanding of its biological efficacy and molecular mechanisms in protecting against CD‐induced oxidative damage.

So future research should focus on exploring the interaction mechanisms of PC‐NL with intestinal cells and the microbiota, and developing novel surface modification strategies and smart delivery systems to enhance its targeting and bioavailability. Furthermore, in vivo studies and systematic pharmacokinetic evaluations are necessary to clarify its functional mechanisms. Thereby future studies will ultimately provide a theoretical foundation for developing PCNL‐FD as a functional food or nutritional intervention for celiac disease and advancing its clinical application.

## 5 Conclusion

This study found that 4% trehalose as a cryoprotectant for PC‐NL effectively maintained liposomal encapsulation efficiency and post‐rehydration stability. Additionally, PCNL‐FD exhibited excellent antioxidant activity and sustained release properties in both in vitro antioxidant assays and simulated digestion experiments. In in vitro experiments, PCNL‐FD significantly reduced ROS levels and MDA content in CD model cells while increasing GSH levels and enhancing SOD and CAT activity. Moreover, PCNL‐FD lowered pro‐inflammatory cytokines TNF‐α and IL‐6 while increasing anti‐inflammatory cytokines IL‐4 and IL‐10. Furthermore, PCNL‐FD regulated the Keap1/Nrf2 signaling pathway, upregulating NQO‐1 and HO‐1 expression, thereby mitigating oxidative stress‐induced cellular damage. The nanoliposome delivery system significantly improved the bioavailability and stability of PC, overcoming its poor water solubility and susceptibility to degradation. Consequently, the antioxidant and anti‐inflammatory effects of PCNL‐FD were superior to those of free PC, providing valuable insights into the potential application of PCNL‐FD for oxidative damage intervention in inflammatory diseases such as CD. These findings suggest that PCNL‐FD could be a promising intervention strategy for CD.

## Author Contributions

**Zhou Ning:** data curation (equal), formal analysis (equal), investigation (equal), methodology (equal), writing – original draft (equal). **Ren Hongtao:** supervision (equal), project administration (equal), writing – review and editing (equal). **Chen Linlin:** data curation (equal), investigation (equal), methodology (equal). **Zhang Xing:** data curation (equal), formal analysis (equal), investigation (equal). **He Kunmiao:** investigation (equal), methodology (equal). **Xu Tiantian:** data curation (equal), investigation (equal). **Wang Hanshuo:** data curation (equal), investigation (equal). **Sun Jie:** investigation (equal), methodology (equal). **Yu Miao:** investigation (equal), methodology (equal). **Wang Na:** conceptualization (equal), funding acquisition (equal), project administration (equal), resources (equal), supervision (equal), writing – original draft (equal), writing – review and editing (equal).

## Conflicts of Interest

The authors declare no conflicts of interest.

## Data Availability

Data will be made available on request.
